# Multi-region minutiae depth value-based efficient forged finger print analysis

**DOI:** 10.1371/journal.pone.0293249

**Published:** 2023-11-16

**Authors:** M. Baskar, Renuka Devi Rajagopal, PRASAD B. V. V. S., J. Chinna Babu, Gabriela Pajtinková Bartáková, T. S. Arulananth

**Affiliations:** 1 Department of Computing Technologies, School of Computing, College of Engineering and Technology, SRM Institute of Science and Technology, Kattankulathur, Chengalpattu, Tamilnadu, India; 2 School of Computer Science and Engineering, VIT University, Chennai, Tamilnadu, India; 3 School of Engineering (CSE), Anurag University, Hyderabad, India; 4 Department of Electronics and Communication Engineering, Annamacharya Institute of Technology and Sciences, Rajampet, AP, India; 5 Faculty of Management, Comenius University in Bratislava, Bratislava, Slovakia; 6 Department of Electronics and Communication Engineering, MLR Institute of Technology, Hyderabad, India; Chitkara University, INDIA

## Abstract

The application of biometrics has expanded the wings to many domains of application. However, various biometric features are being used in different security systems; the fingerprints have their own merits as it is more distinct. A different algorithm has been discussed earlier to improve the security and analysis of fingerprints to find forged ones, but it has a deficiency in expected performance. A multi-region minutiae depth value (MRMDV) based finger analysis algorithm has been presented to solve this issue. The image that is considered as input has been can be converted into noisy free with the help of median and Gabor filters. Further, the quality of the image is improved by sharpening the image. Second, the preprocessed image has been divided into many tiny images representing various regions. From the regional images, the features of ridge ends, ridge bifurcation, ridge enclosure, ridge dot, and ridge island. The multi-region minutiae depth value (MRMDV) has been computed based on the features which are extracted. The test image which has a similarity to the test image is estimated around MRMDV value towards forgery detection. The MRMDV approach produced noticeable results on forged fingerprint detection accuracy up to 98% with the least time complexity of 12 seconds.

## 1. Introduction

Various organizations have used the development of information technology to meet their goals. As the organizations have a variety of information on their system, which belongs to different users and business partners, they are responsible for securing the data most effectively. Any organization faces various challenges against the data maintained through threats. The security measures which can be different are enforced to secure the data and handle the problem of illegal access. Access restriction is the most dominant one, which restricts the illegal user from accessing the available data. In this way, different approaches are used, like profile-based access and key-based access restriction methods. However, the performance of such methods is not efficient in meeting the system’s security requirements as they can be tampered with easily by various adversaries. Using biological features is more effective in enforcing such security systems. The facial features and thumb features are more challenging for the adversary that can support such security systems. Fingerprints and palm prints can be used towards the problem effectively.

Human fingerprint has great independence among other features of biometrics. It has unique characteristics which vary between any number of users. It has components of Minutiae ending, bifurcation, islands, dots, and so on. These components can be common in all human fingerprints but vary in numbers and sizes. The components and their numbers can be obtained by processing the fingerprint image. These numbers will not correlate with any other numbers. So, by adopting such finger analysis in security systems, the performance of authentication and illegal access restriction can be enforced most strictly.

**Fig 1:**
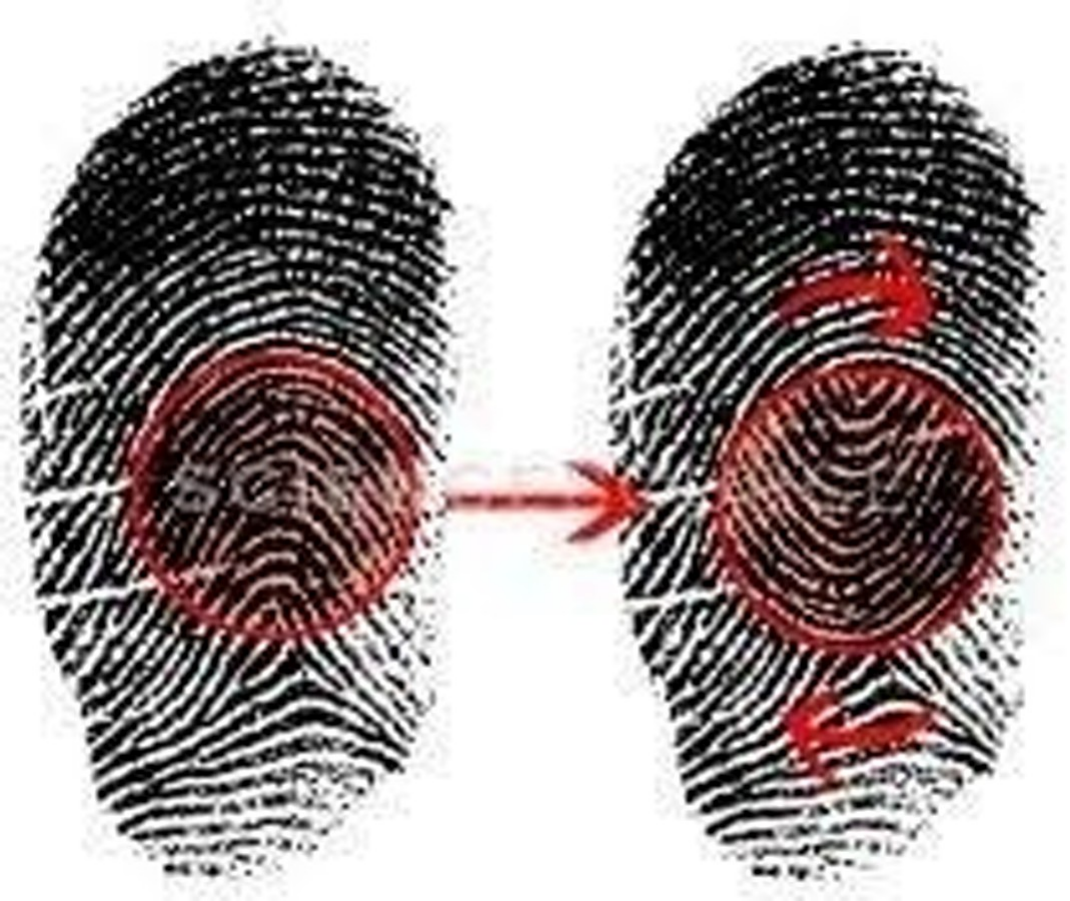
Sample altered fingerprints.

The picture of the sample fingerprint is presented in Fig 1, which has both original and altered fingerprints. The adversary or malformed user would try to breach the security walls by producing an altered print to the system. However the system should be capable of differentiating the original and altered one. So, the security system should consider various features from the ridge like dots, islands, ends, enclosure, and bifurcation. By considering such features in the authentication and verification process, the problem of forgery detection can be handled effectively.

Adapting finger print analysis to organizational security is a highly required process. Because finger print is the most unique feature for any human, and by including such feature for security reasons improves the security of any organization. However, there are adversaries who would produce forged finger print for the system and the system should be more rigid in detecting such forged finger prints. If the system is highly efficient in detecting forged finger prints then the security performance of the system will be highly efficient. This would be used in many security applications from banks to the defense sector.

This score presents an efficient Multi Region Minute Depth Value-Based Efficient Forged Finger Print Analysis model in this article. The model considers the depth of each feature in different regions to perform the verification process. The model estimates the Minutiae Depth Value (MDV) according to the number of features present and their instances with the mass value. By computing such MDV values for various regions, the method computes MRMDV values to classify the fingerprint. The multi-region minutiae depth value (MRMDV) has been computed based on the features which are extracted.

Unlike other methods, the proposed MRMDV based scheme consider various features like ridge ends, ridge bifurcation, ridge enclosure, ridge dot, and ridge island being extracted from various regions of the image. By extracting features from various region and concerning various regional features, the localization of the features are obtained accurately and supports the performance development in forgery detection.

### 1.1 Highlights and problem statement

➢ Organizational security has been approached by using finger print analysis.
➢ Forged finger print detection plays vital role in authenticating the persons in the organization.
➢ The existing methods consider a minimum set of features in detecting forgery in finger print, which in turn produces a higher false ratio.
➢ The previous methods do not concern the entire features of finger print towards forgery detection.
➢ The forgery detection is approached with the multi-region minutiae depth value (MRMDV) based approach.
➢ The method considers regional features of finger print images and supports the achievement of higher accuracy in forgery detection.

The article has been organized to present the introduction, problem statement, highlights and motivation in section 1. Section 2 details the objective of the paper and Section 3 presents the detailed literature survey of the problem and Section 4 presents the contribution of the paper. Section 5, details the complete working model of the proposed system. Section 6 presents the experimental results obtained from the proposed method and presents the comparative study. Section 7, presents the conclusion of the proposed work.

## 2. Objectives

The problem of forgery detection in the fingerprints is analyzed and studied well. The presence of forgery has been identified according to the minutiae features. Any minutiae contain many edges, junctions, bifurcation, and so on. Different methods are available in the literature that consider just the ridge end, edge, dot, enclosures, and bifurcations. But there is no such method that considers the maximum possible features in measuring the similarity towards classification. On the other side, the classification approaches consider the entire print and measure the similarity among the features. But the noise generated by the capturing device or any scare generated on the finger of any person would generate missing features. This introduces higher false classification and affects the performance of classification. This encourages the author to design an efficient approach towards forged fingerprint detection.

## 3. Related work

This section details various methods of fingerprint analysis for forged print detection.

In [1], a fingerprint classification approach is discussed with the use of SVM. The method has been designed to enforce access restriction and verification in crime departments. The method has improved the verification performance.

In [2], a fake fingerprint detection approach is presented, which uses minutiae feature distribution to detect the fake prints according to the orientation features. Similarly, in [3], the author introduced an efficient altered print detection that uses a crossing number minute extraction algorithm. The method splits the images into sectors to extract the features in measuring the similarity between them.

In [4], the analysis of the performance evaluation of various altered fingerprint detection schemes is discussed to present the comparative study. The performance of different fingerprint analysis approaches is evaluated by generating fingerprints artificially to support various research sectors [5]. Such generated data set has been validated by different approaches in [6]. Similarly, an orientation-based approach is presented in [7] towards the problem.

In [8], they analyze how the biometric features obtained from fingerprints are used in access restriction in different control systems. Also, the author presents a comparative study on various fake print detections. In [9], a neuro-fuzzy-orient approach is presented [9], which generates a number of fuzzy rules from the image data set to support classification.

In [10], the author presents a detailed analysis of various fingerprint analysis schemes and fake print detection schemes. Also, a taxonomy of approaches is generated. Similarly, a texture-based approach to fake print detection is presented in [11], which generates a co-occurrence matrix based on the gradient features toward classification.

In [12], a counter-measure-based spoofing attack detection scheme with an altered fingerprint is discussed. Similarly, a CNN model is presented in [13] for the detection of the liveliness of fingerprints. The method extracts the feature distribution by segmenting the image and estimates distribution measures to perform detection. The presence of a spoofed attack is handled with the quality features in [14], which consider the Gabor feature, ridge frequency, and so on. Such features are used in the classification. The features discussed above have been used to evaluate different data sets in [15].

A divide and conquer-based minutiae matching is sketched in [16] to detect fake fingerprints. The method generates sub-samples and matches them with the minutiae using the template matching algorithms.

In [17], the author presented a recognition system that acquires the fingerprint and preprocesses the image by binarization. The feature from the binarized image is extracted using a minutiae extractor to collect the ridge edge and bifurcation. According to the features extracted, feature matching and recognition are performed. The verification is performed by measuring the distance among the minutiae.

In [18], a detailed skeleton of CNN-based deep learning framework for crime scene detection is presented, which uses the data sets that contain several photographs that are incomplete, and the method extracts the minutiae and classifies them according to the CNN available. In [19], a fake print analysis approach is presented, which analyzes different images obtained from different sensors. The images from optical and thermal sensors are obtained, and by using the Min-max approach, the normalization is performed. From the normalized images, the method extracts GLCM (Gray Level Co-Occurrence Matrix) image features and is classified with KNN and SVM.

In [20], the author designed an efficient technique to detect forged fingerprint images. The recognition is performed with the Boltzmann machine. The problem of fake fingerprint detection is approached with a Fuzzy Inference System (FIS) with ANN [21] to improve the classification performance. In the Research [21] the authors established a region centric based RCMPM finger print recognition approach. Similarly, a touch-based biometric authentication system is presented for forgery attacks [22]. The method performs authentication by using a behavior-based approach to find the secret values. A rapid forgery detection scheme is presented in [23], which identifies the dissimilar blocks between the forged one and the normal one. The method computes the dissimilarity between the prints by searching the blocks in forged ones. Euclidean measures are computed towards classification, where the images are applied with morphological operation and binarization.

This paper [24] presents a methodology for quantifying heart rate using a fingertip and Arduino microcontroller. The technique is founded upon the principle of Photo Plethysmography (PPG), a non-invasive approach for quantifying changes in tissue blood volume employing a light source and detector. During cardiac activity, the heart facilitates blood circulation throughout the entire body, resulting in fluctuations in blood volume within the finger artery. The detection of blood fluctuation can be achieved by employing an optical detecting mechanism near the fingertip. The signal is capable of being amplified and afterward transmitted to an Arduino microcontroller through the utilization of serial port connectivity. Heart rate monitoring and counting are conducted using data processing technology.

This study [25] introduces a novel approach for detecting and eliminating shadows, explicitly addressing the issue of counterfeit shades. The proposed method leverages the HOG (Histogram-of-Oriented Gradients) characteristics to achieve effective results. During the initial phase of moving object identification, the Gaussian Mixture Model (GMM) is employed to segregate the foreground regions accurately. Using the HSV color space enhances the differentiation between chromaticity and intensity, hence facilitating the identification of shadows within the fragmented context. However, it is essential to note that this approach may inadvertently misclassify certain portions of objects as shadow regions. The utilization of the observed phenomenon that object regions, specifically incorrectly categorized places and fake shadows, significantly alter the background data. This contrasts casting shadows, which primarily induce intensity variations across the background. They employ a local feature-matching technique to distinguish between genuine and counterfeit shadow regions accurately. Once the areas have been determined, it becomes feasible to eliminate the shadows present in the object regions without compromising any information. The experimental findings demonstrate that the suggested approach yields favorable outcomes in outdoor environments.

The SVM based approach [1], considers only the limited features in the classification of finger print. The crossing minutiae extraction algorithm used in [3], generates sectional features in the detection of fake finger print, however considering only the minutiae features only. This introduces poor accuracy in the classification. The neuro-fuzzy approach [9], generates fuzzy rules from limited features which restrict the accuracy of fake print detection. The texture-based scheme [9], generates co-occurrence matrix from gradient features alone which leads to poor accuracy in fake print detection. The CNN model presented in [13], uses distributional measures in fake finger print detection, but suffers from poor accuracy as the feature distribution will vary on age. The FIS-ANN model [21], perform forgery detection according to region features but lacks to consider entire features [26–29]. All the above-discussed methods are not efficient in detecting fake fingerprints, which raises the requirement for efficient techniques to be modeled [30–32].

## 4. Contributions

➢ Towards maximizing the performance of forged fingerprint detection, a novel scheme is designed and discussed in this section.
➢ Unlike other methods, the proposed MRMDV model considers all possible features of the finger print.
➢ The method considers the features in various regions towards finger print analysis.
➢ Based on the features collected, the method computes MRMDV value against various features to support localization and classification.
➢ The method introduces higher accuracy in classification with less false ratio.

## 5. Methodology

The multi-region minutiae depth value-orient scheme applies a multi-level Gabor filter used to remove the noise from the given input image and thereby increase in the image quality. Further, the image has been cropped into tiny regions as per the size of the window that is considered for the evaluation. The features from each tiny regional image are extracted according to the features considered. By using the features that are extracted, this method can be used to estimate the MDV value to compute the MRMDV value to support the classification process.

**Fig 2:**
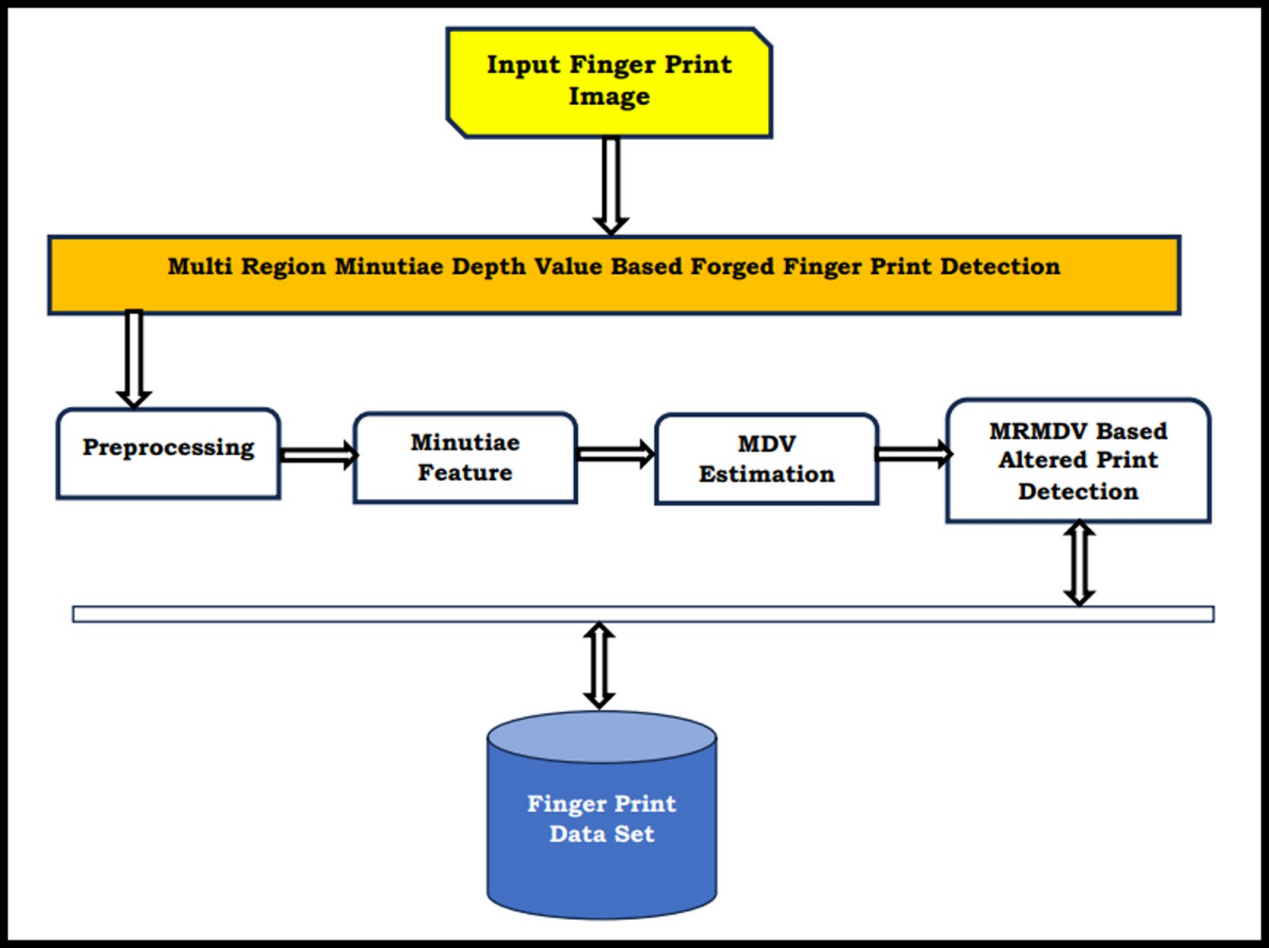
Structure of MRMDV-based forged finger print detection.

Fig 2 shows the functional diagram of the MRMDV approach, and each component is discussed in this section.

### 5.1 Preprocessing

The image given has been applied with median and Gabor filters to remove the noise particles generated at capturing. The image, which is eliminated from the noise, is improved for its sharpness to enhance the image quality. The image with good quality is used in extracting the features of the image to support fake fingerprint detection.

**Algorithm:**
Given: Finger Print FP_Img
Obtain: Quality Improved image Qimg
Begin
  Fetch Fpimg
    
$$\text{Gabor}\;\text{Filter}\;\text{GF}\begin{array}{c} NL\\ =\;Initialize\;(GF,\;Coefficients\;).\\ i=1 \end{array}$$
  At all levels l
      
Qimg=GF(I,FPImg)
  At_End
Stop

The preprocessing approach applies the Gabor filter to eliminate the noisy parts and improve the image quality.

### 5.2 Minutiae feature extraction

The features of minutiae from the fingerprint image are extracted here. To perform this, the image is divided into a number of tiny images. By considering specific window size, the method crops the image into a number of regions. From the region images generated, the method extracts the concerned features. At each tiny part, the features of bifurcation, minutiae dots, Minutiae Island, ends, and enclosures are extracted. Obtained features are framed to form a feature vector to be utilized to compute MDV value.

**Algorithm:**
Given: Quality Improved Image Qimg
Obtain Feature Vector Set Fvs.
Begin
  Fetch Quality Improved Image Qimg
  Split image into regional image.
    
$${\text{RI}=ò}_{\text{i=1}}^{\text{Nos}}\text{split(EI,<}\;Sa\text{,}\;Ea>$$
  Nos–no of sections or regions
  Sa–Starting angle
  Ea- Ending angle.
  From all regional images, Ri
     
$$\text{Obtain}\;\text{Minutiae}\;\text{Island}\;\text{Miland}=\begin{array}{c} Size(Ri)\\ \left[Islands\exists Ri\right]\\ i=1 \end{array}$$
     
$$\text{Obtain}\;\text{dots}\;\text{dot}=\begin{array}{c} Size(Ri)\\ \left[Dots\exists Ri\right]\\ i=1 \end{array}$$
     
$$\text{Obtain}\;\text{Ends}\;\;\text{Ed}=\begin{array}{c} Size(Ri)\\ \left[Ends\exists Ri\right]\\ i=1 \end{array}$$
     
$$\text{Obtain}\;\text{Enclosures}\;\text{Ens}=\begin{array}{c} Size(Ri)\\ \left[Enclosures\exists Ri\right]\\ i=1 \end{array}$$
     
$$\text{Obtain}\;\text{Bifurcation}\;\text{Bfn}=\begin{array}{c} Size(Ri)\\ \left[Bifurcation\exists Ri\right]\\ i=1 \end{array}$$
     
GeneratefeaturevectorFv={Miland,dot,Ed,Ens,Bfn}
     
Fvs=∑(Fv∈Fvs)∪Fv
Stop

The features of the finger image are extracted according to the minutiae in terms of bifurcation, enclosure, dot, island, and ends. The features obtained are converted to feature vectors in estimating MDV values.

### 5.3 MDV estimation

The Minutiae depth value (MDV) of any fingerprint shows the containment of mass ridge features. The value of MDV has been measured according to the frequency of dots, edges, bifurcation, ends, and enclosures. To measure the MDV value, the method counts the occurrence of all the features and estimates the frequency of each feature to compute the value of MDV. With the MDV value measured, the method performs the detection of altered print.

**Algorithm:**
Given: Feature Vector Fv
Obtain: MDV
Begin
  Fetch Fv.
    
$$\text{Count}\;\text{Occurrence}\;\text{of}\;\text{dots}\;\text{dc}=\int_{\text{i}=1}^{\text{size(Fv})}Count(Fv(i)\;type\;==dot$$
    
$$\text{Count}\;\text{occurrence}\;\text{of}\;\text{Edge}\;\text{Ec}=\int_{\text{i}=1}^{\text{size}(\text{Fv})}Count(Fv(i)\;type\;==edge$$
    
$$\text{Count}\;\text{Occurrence}\;\text{of}\;\text{ends}\;\text{Enc}=\int_{\text{i}=1}^{\text{size(Fv})}Count(Fv(i)\;type\;==End$$
    
$$\text{Count}\;\text{Occurrence}\;\text{of}\;\text{enclosure}\;\text{Encc}=\int_{\text{i}=1}^{\text{size(Fv})}Count(Fv(i)\;type\;==Enclosure$$
    
$$\text{Count}\;\text{Occurrence}\;\text{of}\;\text{bifurcation}\;\text{Bc}=\int_{\text{i}=1}^{\text{size(Fv})}Count(Fv(i)\;type\;==\;bifurcation$$
    
$$\text{Measure}\;\text{Minute}\;\text{Depth}\;\text{Md}=\frac{Encc}{size(SI)}\times \frac{dc}{encc}\times \frac{ec}{bc}$$
    
$$\text{Measure}\;\text{Minute}\;\text{Depth}\;\text{Value}\;\text{MDV}=\frac{\sum MD}{size(Fv)}$$
Stop

The MDV estimation function measures the minutiae depth value according to the occurrence and frequency of all the features considered. The MDV value represents the similarity of the features on specific region of the image. This will be measured for all the regions of the image according to the region image generated and used to measure the final MRMDV value to support the classification.

### 5.4 MRMDV based altered finger print detection

The multi-region approach to fake fingerprint detection is conducted by measuring minutiae depth value for various regions of the print given. To handle this, first preprocessing is performed, which eliminates the noise and improves the quality of the image. Second, regional images are generated, and concern features are obtained. Third, from each regional feature obtained, the value of MDV is measured. Using the MDV value of all the regions, the value of MRMDV is measured to classify the fingerprint given.

**Algorithm:**
Given: Fingerprint image Fpi, Data set Ds.
Obtain: Boolean
Begin
  Read Input image Fpi
  Read data set Ds.
  PI = Preprocessing (Fpi)
  Fv = Perform Minutiae feature extraction (PI)
  From All Feature vector instance Fvi
    MDV_fvi_ = Estimate MDV (Fvi)
  End
    
$$\text{Compute}\;\text{MRMDV}=\frac{\sum MDV}{size(Fvs)}$$
    With any feature vector of trained set
     Measure Minutiae Depth Similarity
  MDS = Dist(MRMDV,MRMDV(ti)
    End
      
$$\text{Compute}\;\text{cumulative}\;\text{MDS}\;\text{CMDS}=\frac{\sum MDS}{size(Ds)}$$
    If CMDS>Th, then
     Alert Altered print.
    End
  End
Stop

The multi-region minutiae depth measure-based approach computes the minutiae depth value on each region which is classified according to the threshold value towards altered fingerprint.

## 6. Experiments and results

The multi-region minutiae depth value-based altered fingerprint detection scheme is hard coded with Matlab Tool, and the performance of the method is evaluated on different parameters. The results obtained are presented in this section.

**Fig 3:**
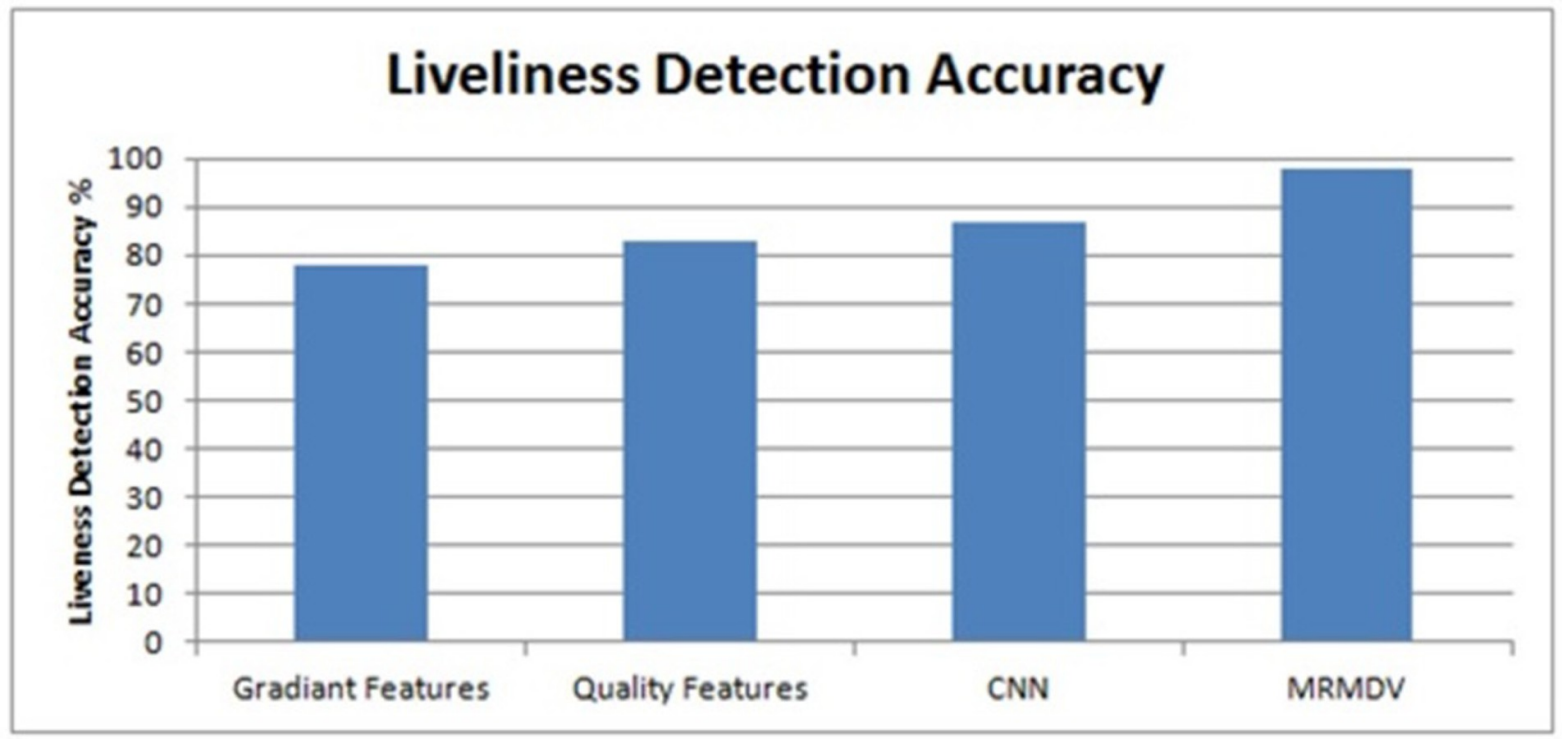
Analysis of liveliness detection accuracy.

The experimental setup being used for the performance evaluation of the proposed MRMDV-based approach is shown in Table 1. The evaluation is conducted by measuring different performance metrics and discussed here. The analysis of liveliness detection accuracy is presented in Fig 3.

**Table 1:** Evaluation details.

Parameter	Value
Tools Used	Matlab
Users	500
Total fake prints	50

The accuracy in liveliness detection is measured for different methods and analyzed in Fig 3. The proposed MRMDV has achieved higher accuracy than other schemes.


$$\;False\;Detection\;Ratio\;=\frac{\;Number\;of\;false\;detetion}{\;Total\;number\;of\;samples}\times 100$$ (1)

Average Classification Error(ACE) which is an averaged sum of APCER and BPCER.

APCER and BPCER can be determined using the Eqs 2 and 3.


$$\text{AP}\;\text{CER}\;=\frac{\;\text{Number}\;\text{of}\;\text{mis}\;-\;\text{classified}\;\text{fake}\;\text{samples}}{\text{Total}\;\text{fake}\;\text{samples}}\times 100$$ (2)

$$\text{BPCER}\;=\frac{\text{Number}\;\text{of}\;\text{mis-classified}\;\text{live}\;\text{samples}}{\text{Total}\;\text{live}\;\text{samples}}\times 100$$ (3)

$$\text{ACE}=\frac{APCER+BPCER}{2}$$ (4)

**Fig 4:**
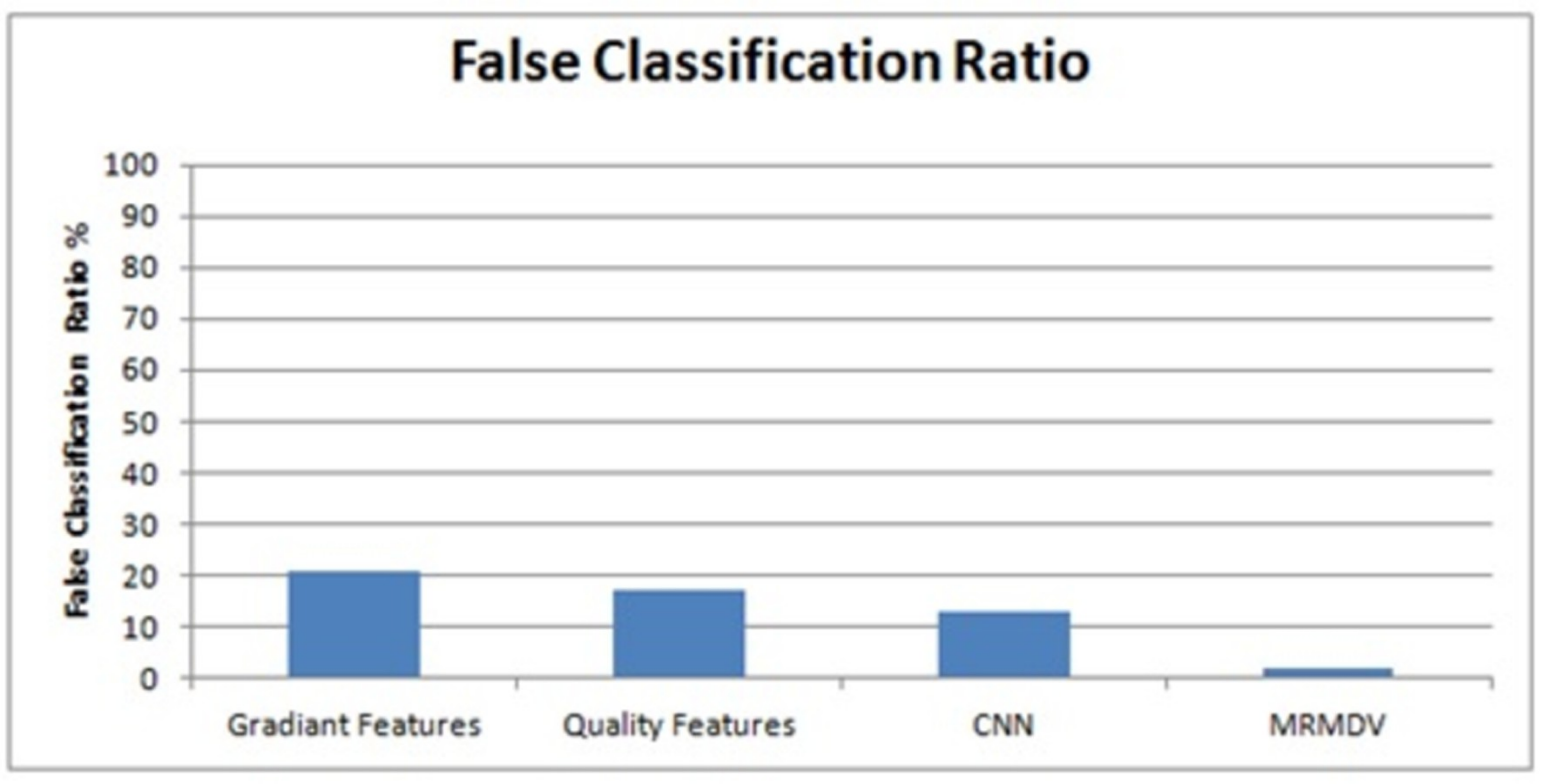
Analysis on false ratio.

**Fig 5:**
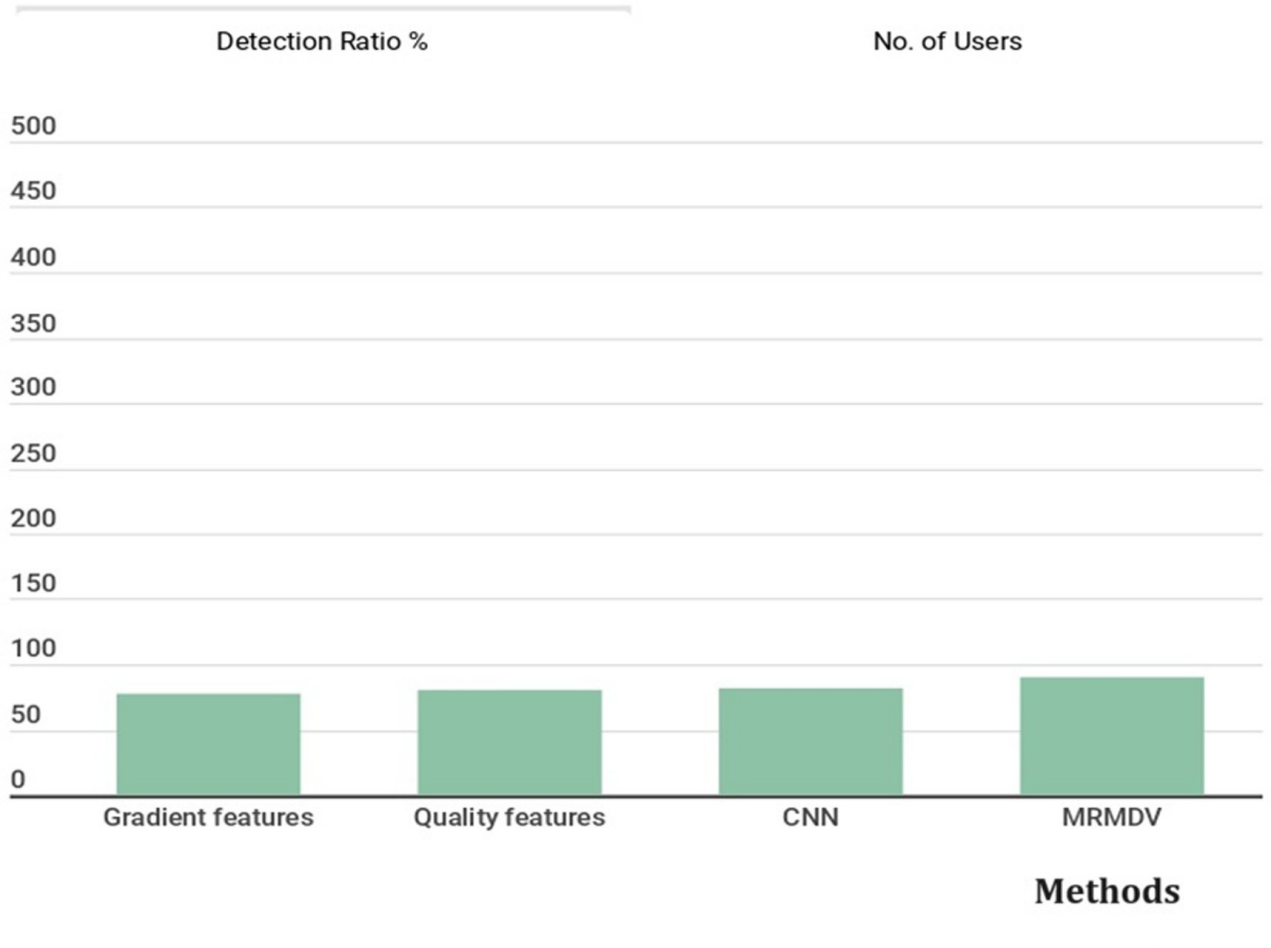
Detection ratio of the uniform users.

The false ratios introduced by different approaches are plotted in Fig 4, where the MRMDV scheme reduces the false ratio. The detection ratio for the uniform users is presented in Fig 5. This provides detailed analysis of uniform users and its related methods.

**Fig 6:**
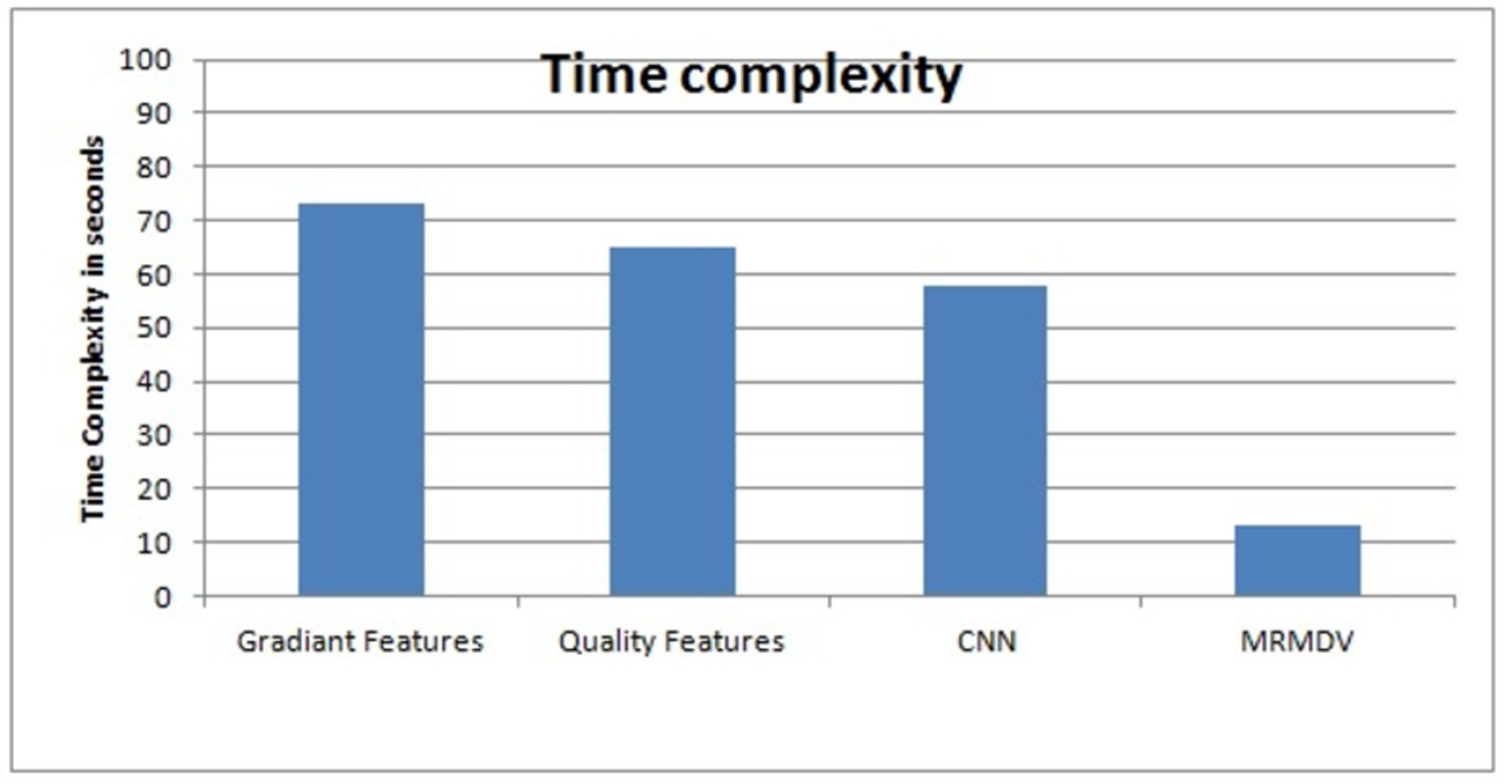
Analysis on time complexity.

The analysis of time complexity produced is as shown in Fig 6. The analysis of time complexity produced and its time complexity measures include various approaches is also presented Fig 6. This figure represents that the proposed MRMDV algorithm introduces less time complexity compared other existing methods considered in this research.

## 7. Discussion and conclusion

In this work, a multi-region minutiae depth based fingerprint analysis algorithm is presented. The input image has been fetched, and noise has been removed to improve the quality of the image. Further, a list of tiny images is cropped from the input image, and features of such tiny images are extracted. The features extracted have been used to measure the value of MRMDV. With the MRMDV value, the value of CMDS is measured to classify the fingerprint as forged or original. The MRMDV scheme achieves higher performance in detecting altered prints with the least time complexity and false ratio.

The proposed MRMDV model has certain limitations on the accuracy. The accuracy is depending on the volume of training set used. Also, the work can be extended by concerning the features like number of rises on edges and number of sharp edges present in the image towards measuring the similarity.

## Supporting information

S1 File Code for preprocessing and edge detection. (DOCX)
