# Supplementary material for: Multi-region minutiae depth value-based efficient forged finger print analysis
Source: PLoS One. 2023 Nov 16;18(11):e0293249. doi: 10.1371/journal.pone.0293249 (PMC10653521; doi:10.1371/journal.pone.0293249)
Supplement: S1 File — (DOCX) [file pone.0293249.s001.docx]

**Code for Preprocessing and Edge detection**

import cv2

import numpy as np

from matplotlib import pyplot as plt

img = cv2.imread(chinna.jpg)

edges = cv2.Canny(img,100,200)

plt.subplot(121),plt.imshow(img,cmap = &#39;gray&#39;)

plt.title(Original Image), plt.xticks([]), plt.yticks([])

plt.subplot(122), plt.imshow(edges,cmap = 39;gray39;)

plt.title(“Edge Image”), plt.xticks([]), plt.yticks([])

plt.show()

cv2.imsave(“edges.jpg")

Code for Minutiae Feature Extraction:

import cv2

import numpy as np

from matplotlib import pyplot as plt

import fingerprint_feature_extractor

def Feature_Extraction(fname)

img = cv2.imread(fname, 0)

[FeaturesTerminations, FeaturesBifurcations , Miland,dot,Ed,Ens]= fingerprint_feature_extractor.extract_minutiae_features(img, spuriousMinutiaeThresh=10, invertImage=False, showResult=True, saveResult=True)

Code for MDV estimation:

Import cv2

Import numpy as np

Def MDV_Estimation(FeaturesTerminations, FeaturesBifurcations , Miland,dot,Ed,Ens)

Med=0

For i=1:size

Cdot = count(dot)

Ced = Count(Ed)

Cens = Count(Ens)

Cbf = Count(FeaturesBifurations)

Cmiland = Count(Miland)

Md = (Cens/Ced)×(Cdot/Ced)×(Ced/Cbf)

Med = Med+md

end

Mdv = Med/size

Code for Foregery Detection:

Import cv2

Import numpy as np

Imgs = list(directory)

Mrmdv=0

For i=1:size

PI = Proprocessing(imgs(i).fname)

[FeaturesTerminations, FeaturesBifurcations , Miland,dot,Ed,Ens ]= Feature_Extraction(PI)

MDV = MDV_Estimation(FeaturesTerminations, FeaturesBifurcations , Miland,dot,Ed,Ens )

Mrmdv = mrmdv+MDV

End

Mrmdv =mrmdv/size

For i=1:size

MDS=Dist(mrmdv,mrmdv(i) )

Cmds = cmds+MDS

End

cmds= cmds/size

if cmds>th then

print(“Alert Altered print”)

End
